# Mortality and mode of dialysis: meta-analysis and systematic review

**DOI:** 10.1186/s12882-023-03435-4

**Published:** 2024-01-03

**Authors:** Subhash Chander, Sindhu Luhana, FNU Sadarat, Om Parkash, Zubair Rahaman, Hong Yu Wang, FNU Kiran, Abhi Chand Lohana, FNU Sapna, Roopa Kumari

**Affiliations:** 1 Department of Medicine, Icahn School of Medicine at Mount Sinai, New York, USA; 2 Department of Medicine, AGA khan University Hospital, Karachi, Pakistan; 3 Department of Medicine, University at Buffalo, New York, USA; 4 Department of Medicine, Montefiore Medical Centre, Wakefield, New York, USA; 5 Department of Pathology, Northwell Health Staten Island University Hospital, New York, USA; 6 Department of Medicine, WVU, Camden Clark Medical Centre, Parkersburg, WV USA; 7 Department of Pathology, Albert Einstein School of Medicine, Montefiore Medical Centre, New York, USA; 8 Department of Pathology, Icahn School of Medicine at Mount Sinai, New York, USA

**Keywords:** Hemodialysis, Peritoneal dialysis, CRRT, Continuous kidney replacement therapy

## Abstract

**Background:**

The global use of kidney replacement therapy (KRT) has increased, mirroring the incidence of acute kidney injury and chronic kidney disease. Despite its growing clinical usage, patient outcomes with KRT modalities remain controversial. In this meta-analysis, we sought to compare the mortality outcomes of patients with any kidney disease requiring peritoneal dialysis (PD), hemodialysis (HD), or continuous renal replacement therapy (CRRT).

**Methods:**

The investigation was conducted according to Preferred Reporting Items for Systematic Reviews and Meta-Analyses (PRISMA). PubMed (MEDLINE), Cochrane Library, and Embase databases were screened for randomized trials and observational studies comparing mortality rates with different KRT modalities in patients with acute or chronic kidney failure. A random-effects model was applied to compute the risk ratio (RR) and 95% confidence intervals (95%CI) with CRRT vs. HD, CRRT vs. PD, and HD vs. PD. Heterogeneity was assessed using *I*^2^ statistics, and sensitivity using leave-one-out analysis.

**Results:**

Fifteen eligible studies were identified, allowing comparisons of mortality risk with different dialytic modalities. The relative risk was non-significant in CRRT vs. PD [RR = 0.95, (95%CI 0.53, 1.73), *p* = 0.92 from 4 studies] and HD vs. CRRT [RR = 1.10, (95%CI 0.95, 1.27), *p* = 0.21 from five studies] comparisons. The findings remained unchanged in the leave-one-out sensitivity analysis. Although PD was associated with lower mortality risk than HD [RR = 0.78, (95%CI 0.62, 0.97), *p* = 0.03], the significance was lost with the exclusion of 4 out of 5 included studies.

**Conclusion:**

The current evidence indicates that while patients receiving CRRT may have similar mortality risks compared to those receiving HD or PD, PD may be associated with lower mortality risk compared to HD. However, high heterogeneity among the included studies limits the generalizability of our findings. High-quality studies comparing mortality outcomes with different dialytic modalities in CKD are necessary for a more robust safety and efficacy evaluation.

## Key learning points

**What is already known about this subject**

End-stage renal disease (ESRD) is a significant public health issue with high mortality rates. Dialysis is the most common form of treatment for ESRD patients, and the choice of dialytic modality can have an impact on patient outcomes.

**What this study adds**

Since previous studies have been inconclusive regarding patient outcomes, this study provided an updated meta-analysis on mortality outcomes with dialytic modalities. Also, unlike most previous meta-analyses, we included randomized trials and observational studies comparing mortality outcomes with dialytic modalities irrespective of the type of kidney disease.

**What impact this may have on practice or policy**

This study indicates that the current evidence is insufficient to compare the safety of dialytic modalities. Well-powered multi-centered trials are required to inform clinical practice better.

## Introduction

Kidney disease poses a significant global health burden. In 2017, an estimated 697·5 million individuals worldwide had chronic kidney disease (CKD) (9.1% prevalence) [1]. CKD ranked as the 12th leading cause of global deaths, with CKD attributed as the direct cause of 1.2 million deaths [1]. In addition, another 1.4 million deaths due to cardiovascular disease had underlying impaired kidney function [1]. Although reliable global estimates on the incidence or prevalence of acute kidney injury (AKI) are unavailable, a meta-analysis by Susantitaphong et al. [2] indicated that the incidence may be as high as 21.6% in adults and 33.7% in children, with AKI-associated mortality rates of 23.9% and 13.8%, respectively.

A severe decline in kidney function (GFR < 15 ml/min/1.73 m^2^) due to either AKI [3] or CKD [4] necessitated life-saving support using kidney replacement therapy (KRT). The global use of KRT has increased by 43% from 1990 to 2017 [1], mirroring the global increase in the incidence of AKI [5] and CKD [1]. Multiple KRT modalities are available: Intermittent hemodialysis is typically used in hemodynamically stable patients, while continuous renal replacement therapy (CRRT) and peritoneal dialysis (PD) are used for hemodynamically unstable patients [3]. Recently, hybrid therapies such as sustained low-efficiency dialysis (SLED) and extended-duration dialysis (ED) allow low dialysate and blood flow rates and, therefore, prolonged dialytic duration in critically ill patients [6, 7]. The advantages and disadvantages of dialytic modalities have been previously reviewed in detail [3, 4, 7].

Despite the growing clinical usage, patient outcomes with KRT modalities remain controversial [6]. Several clinical trials [8, 9] and meta-analytic studies [10–13] demonstrate no significant differences in in-hospital mortality, in-ICU mortality, renal recovery, or dialysis dependence between KRT modalities. However, these studies exclusively recruited patients with AKI. In contrast, a meta-analysis by Han et al. [14] reported higher mortality rates with PD than HD in patients with end-stage kidney disease (ESKD). However, the study population was limited to Korean adults aged ≥ 65 years. Similarly, Brimble et al. [15] demonstrated higher mortality risk with higher peritoneal membrane solute transport rate during PD in a pooled population of patients receiving KRT for severe AKI or CKD but did not compare mortality outcomes with other KRT modalities. Failure associated with PD has also been attributed to the peritoneal membrane being an ineffective long-term ultrafiltration membrane for waste removal, primarily due to increased inflammatory responses and peritoneal infection [16].

Furthermore, there is some evidence to indicate that IHD may be inferior to other dialytic modalities in terms of clinical outcomes. For instance, an analysis of Swedish nationwide data on adult general ICU patients with acute kidney failure requiring KRT showed better renal recovery with CRRT than HD with no difference in mortality rates [17]. In addition, CRRT may also produce better cardiovascular stability than HD [18]. Higher systemic oxygen consumption, need for inotropic support, and occurrence of intestinal intramucosal acidosis have been reported during HD [19].

Given these inconclusive findings, we sought to compare the mortality outcomes of patients with any kidney disease (AKI, AKD, or CKD) requiring one of the three broad categories of KRT (HD, PD, and CRRT) in this meta-analysis. Although we included randomized controlled trials (RCT) and observation cohort studies in our analysis, we conducted a sub-group analysis to identify the potential impact of study design on mortality outcomes with KRT.

## Methodology

### Definitions: AKI, AKD, and CKD

Although historically, CKD and AKI were considered separately, there is a growing recognition of the bidirectional risk relationship between CKD and AKI and, in some cases, seen as a continuum of the disease process [20–24]. In its 2020 consensus conference, Kidney Disease: Improving Global Outcomes (KDIGO) stipulated that AKI and CKD “do not constitute a diagnosis” but are descriptors of abnormal kidney structure/function. For instance, AKI is characterized by a rapid decline in kidney function with a 50% increase in serum creatinine (SCr) within seven days or 0.3 mg/dl within two days or oliguria for over six hours [22]. A slower decline in kidney function with AKI, a 50% increase in SCr, glomerular filtration rate (GFR) < 60 mL/min per 1.73 m^2,^ or a decrease in GFR by ≥ 35% is classified as acute kidney disease (AKD) [22]. In contrast, CKD constitutes a gradual decline in kidney function over more than 90 days staged with GFR < 60 mL/min per 1.73 m^2^ [22, 25]. Other markers of kidney damage may include an albumin–creatinine ratio (ACR) ≥ 30 mg/g, urinary sediment abnormalities, abnormalities associated with tubular disorders such as electrolyte imbalance, and abnormalities detected by histology or an imaging procedure [25].

### Study design

This systematic review and meta-analysis were conducted according to the guidelines of the Cochrane methodology and Preferred Reporting Items for Systematic Reviews and Meta-Analyses (PRISMA) [26].

### Literature search

Three databases (PubMed (MEDLINE), Cochrane Library, and Embase) were screened using the following search query: (mortalities OR deaths OR fatalities OR casualties OR “mortality rates” OR “mortality outcomes”) AND (dialysis OR “renal replacement therapy” OR “kidney replacement therapy” OR “blood purification”) OR (hemodialysis OR HD OR “extracorporeal dialysis” OR “blood dialysis”) OR (“continuous renal replacement therapy” OR CRRT OR “continuous dialysis” OR “continuous hemofiltration”) OR (“peritoneal dialysis” OR PD OR “intraperitoneal dialysis” OR “abdominal dialysis”). All studies from inception to December 2022 were considered for screening.

Database searches were conducted in three phases to cover the three comparisons: CRRT versus HD, PD versus HD, and CRRT versus PD. We also screened through the reference lists of several studies, especially previous meta-analyses and systematic reviews covering some of our research objectives.

### Eligibility criteria

The eligibility criteria for this study were developed using the PECO (Participants, Exposures, Comparators, and Outcomes) framework. The participants included patients with AKI, AKD, or CKD requiring any dialytic modality. We did not place age limits on the study participants to increase the scope of the studies covering the three comparisons of the dialysis modalities under investigation. However, all the included studies were required to provide detailed inclusion criteria of the participants, such as age limits, the type of kidney disease (acute or chronic), presenting clinical signs by the patients (uremia, albuminuria, oliguria, etc.), and previous interventions conducted. The exposure and comparator characteristics for the included participants were one of the three modalities for dialysis (CRRT, PD, or HD). Since these modes also have other types and techniques for performing dialysis, the included studies were required to provide these specifications. Lastly, the primary outcome of interest was the mortality rate of patients using different dialysis modes.

Only randomized controlled studies or observational studies published in English with online full-text access options were considered for inclusion. We excluded studies with a high risk of bias, animal studies, conference abstracts, and incomplete or missing outcome data.

### Data extraction

Data from the selected studies were extracted into a pre-defined MS Excel sheet, with primary data points including the author, study design, study duration or dates (start and end date), participant number, participant demographics [age (mean ± standard deviation), and gender (%Male)], inclusion criteria (underlying disease, chronic or acute kidney disease, presenting signs, age categories, etc.), the type of dialysis modalities under investigation, and the mortality rates (% per modality group).

Two independent reviewers extracted data, and discrepancies were resolved through discussion with a third author.

### Quality assessment

Two authors independently evaluated the quality of the included studies using the Cochrane Risk of Bias 2 tool for RCTs. The tool involves assessing seven domains: randomization process (selection bias), concealment of the allocation sequence (selection bias), blinding of participants and health professionals (performance bias), blinding of outcome assessment (detection bias), missing outcome data (attrition bias), selective reporting of results (reporting bias), and other potential sources of bias. Assessment decisions were categorized as ‘low risk of bias,’ ‘high risk of bias,’ or ‘some concerns.’ Any discrepancies between the two authors were resolved through discussion with a third author. We also used funnel plots to check for potential publication bias and the *I*^2^ statistic to detect heterogeneity.

### Statistical analysis

The R software version 4.1.0 (R Foundation for Statistical Computing, Vienna, Austria, 2021) was used to conduct the meta-analysis part of the investigation. A random-effects model was applied to compute the risk ratio (RR) and 95% confidence intervals (95%CI), while the *I*^2^ statistic was used to assess heterogeneity. Heterogeneity was assessed on a scale with 0% as complete consistency and 100% as complete inconsistency, with the *I*^2^ ≤ 50% as the threshold for reliability. A *p*-value of < 0.05 was considered statistically significant. The three outcomes under analysis were the mortality rate comparisons between CRRT vs. HD, CRRT vs. PD, and HD vs. PD.

## Results

### Study selection

**Fig. 1:**
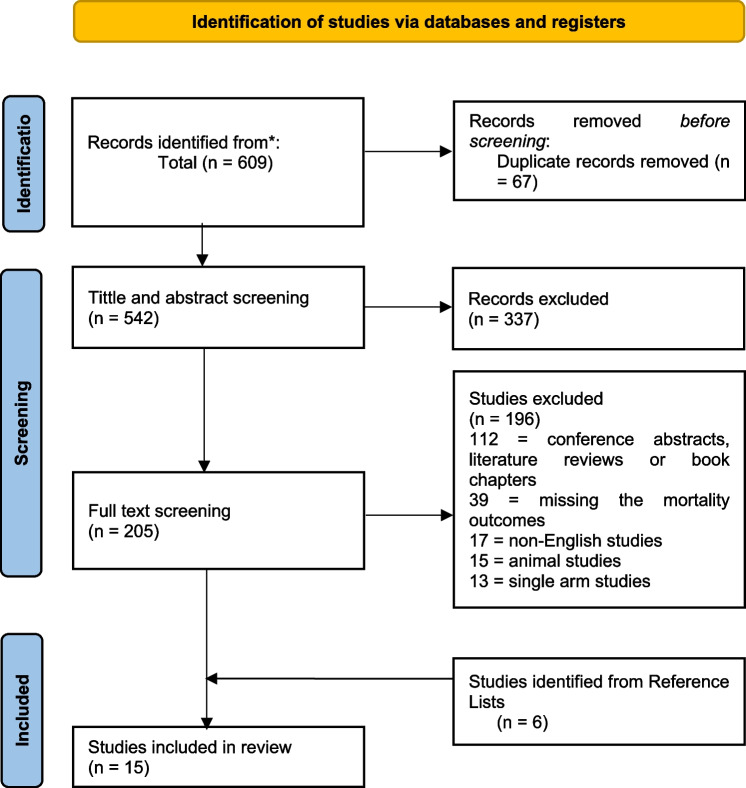
PRISMA flow diagram detailing the study selection process

The detailed PRISMA flowchart of the study selection process is presented in Fig. 1. Briefly, the initial database search resulted in 609 studies, of which 67 were excluded due to duplication. In the title and abstract screening phase, 196 reviews, 21 case reports, 66 literature reviews, and 54 studies that lacked exposure, comparator, or outcome of interest were excluded. Another 196 studies were excluded during full-text screening for not meeting the inclusion criteria. Therefore, the database search yielded nine articles that met our inclusion criteria. Six additional studies were identified from the reference lists of other reviews, bringing the final number of included studies to 15. The characteristics of the included studies are presented in Table 1.

**Table 1:** Characteristics of included studies

Author (Year)	Design	Inclusion criteria	n (% male)	Mean/median age	Duration	Mortality
Continuous Renal Replacement Therapy (CRRT) versus Peritoneal Dialysis (PD)						
Al-Hwiesh et al. [27] (2018)	RCT	• AKI requiring KRT	CRRT = 62 (72.6%) PD = 63 (74.6%)	CRRT = 44.6 ± 12.4 PD = 45.4 ± 4.1	28 days	CRRT = 53.2% PD = 30.2%
George et al. [28] (2011)	RCT	• AKI • No history of abdominal surgery • No pulmonary edema	CRRT = 25 (60%) PD = 25 (64%)	CRRT = 45.3 ± 17.5 PD = 45.3 ± 17.5	3 years	CRRT = 84% PD = 72%
Jaryal et al. [29] (2017)	R-Obs	• > 12 years • GFR < 60 ml/min/1.73m^2^	CRRT = 22 (50%) PD = 18 (66.7%)	CRRT = 55.5 ± 19 PD = 51.1 ± 16	1 year	CRRT = 81.8% PD = 77.8%
Phu et al. [30] (2002)	RCT	• > 15 years • AKI requiring KRT	CRRT = 34 (88%) PD = 36 (75%)	CRRT = 35 (29.5–38.2) PD = 36 (29.6–38.4)	1 year	CRRT = 15% PD = 47%
Peritoneal Dialysis (PD) versus Hemodialysis (HD)						
Basu et al. [31] (2016)	R-Obs	• 1 month to 16 years • Uremia, oliguria, anuria, and severe metabolic acidosis • Non-responsive fluid overload, • Persistent hyponatremia	PD = 84 (51%) HD = 52 (67%)	PD = 3.2 (0.1–7.6) HD = 8.4 (3.2–15.6)	30 days	PD = 39.3% HD = 66.3%
Fenton et al. [32] (1997)	R-Obs	• ESRD • No history of dialysis or kidney transplant	PD = 2841 (NR) HD = 7792 (NR)	NR	5 years	PD = 26.2% HD = 30.9%
Gabriel et al. [33] (2008)	RCT	• With acute tubular necrosis	PD = 60 (72%) HD = 60 (66%)	PD = 62.5 ± 21.2 HD = 64.2 ± 19.8	60 days	PD = 58% HD = 53%
Murphy et al. [34] (2000)	P-Obs	• Patients with renal failure of any kind	PD = 282 (59.9%) HD = 540 (58.7%)	PD = 56.1 (54.2–58.0) HD = 59.4 (58.1–60.7)	6 months	PD = 33.8% HD = 39.8%
He et al. [35] (2020)	R-Obs	• Patient with ESRD	PD = 501 (58%) HD = 436 (53.9%)	PD = 51.69 ± 14.47 HD = 57.11 ± 15.92	5 years	PD = 4% HD = 21%
Liem et al. [36] (2007)	R-Obs	• > 18 years • Requiring > 30 days KRT	PD = 5802 (61.3%) HD = 10,841 (57.5%)	PD = 53.6 ± 15 HD = 61.8 ± 14.6	6 months	Hazard ratio (PD vs. HD) = 0.70 (95% CI 0.67–0.74)
Hemodialysis (HD) versus Continuous Renal Replacement Therapy (CRRT)						
Gaudry et al. [15] (2022)	RCT	• AKI requiring KRT	HD = 274 (63.9%) CRRT = 269 (62.4%)	HD = 66.8 ± 13.3 CRRT = 66.2 ± 13.4	60 days	HD = 46.3% CRRT = 54.3%
Liang et al. [37] (2016)	R-Obs	• No history of chronic kidney dialysis, transplant, or heart failure • Creatinine level < 4 mg/dl	HD = 353 (57.5%) CRRT = 285 (58.6%)	NR	90 days	HD = 55.9% CRRT = 60%
Schefold et al. [38] (2014)	RCT	• > 18 years • AFR requiring KRT	HD = 128 (63.3%) CRRT = 122 (61.5%)	HD = 60.8 ± 13.4 CRRT = 62.3 ± 14.5	30 days	HD = 52.4% CRRT = 45.4%
Truche et al. [39] (2016)	P-Obs	• Patients who received MV for ≥ 48 h in an ICU setting • No history of kidney transplant, CKD requiring KRT, or heart disease	HD = 816 (65%) CRRT = 544 (63.6%)	HD = 66.4 (54.8–76.1) CCRT = 64.3 (52.4–74.3)	30 days	CRRT = 46.5% HD = 35%
Yilmaz Aydin et al. [40] (2022)	P-Obs	• AKI requiring KRT • No history of CKD or ESRD	HD = 80 (38.7%) CRRT = 40 (37.5%)	HD = 61.4 ± 15.2 CRRT = 65.9 ± 9.1	30 days	HD = 66.3% CRRT = 67.5%

*AKI*  acute kidney injury, *ARF *Acute renal failure, *ESRD* end-stage renal disease, *GFR* glomerular filtration rate, *KRT * renal replacement therapy, *MV *mechanical ventilation, *NR * not reported, *P-Obs * prospective observational study, *R-Obs *retrospective observational study

### CRRT vs. PD

**Fig. 2:**
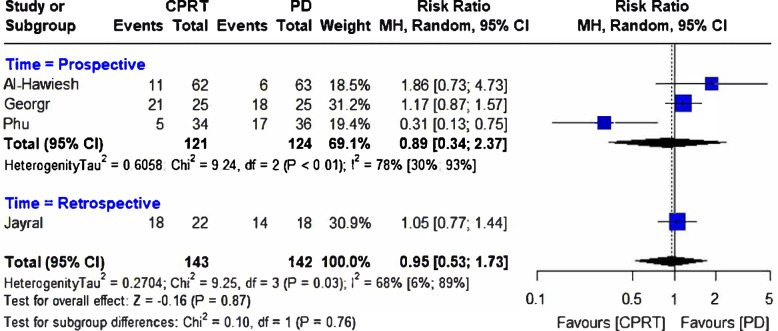
Forest plot comparing mortality rates between continuous renal replacement therapy (CRRT) and Peritoneal Dialysis (PD)

**Fig. 3:**
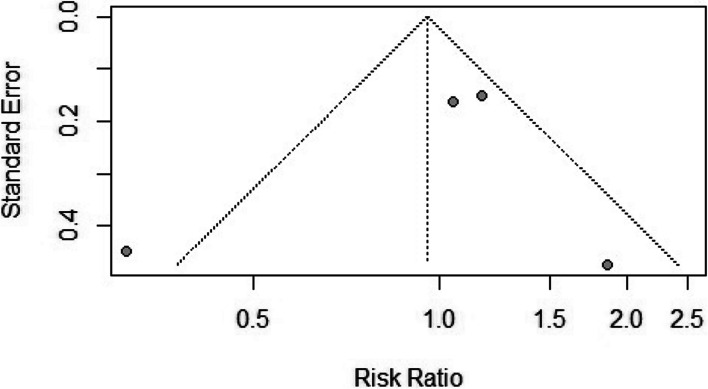
The funnel plot representing the publication bias between the selected studies comparing continuous renal replacement therapy (CRRT) and peritoneal dialysis (PD)

Four selected studies with a pooled participant size of 143 in the CRRT and 142 in the PD groups reported mortality rates [27–30]. There was no difference in the mortality risk between the two groups [RR = 0.95, (95%CI 0.53, 1.73), *p* = 0.92] (Fig. 2). However, the included studies had moderate heterogeneity (*I*^2^ = 68%; *p* = 0.03). The findings remained unchanged when prospective and retrospective studies were separately assessed in the subgroup analysis (Fig. 2). The funnel plot showing the publication bias is presented in Fig. 3.

**Fig. 4:**
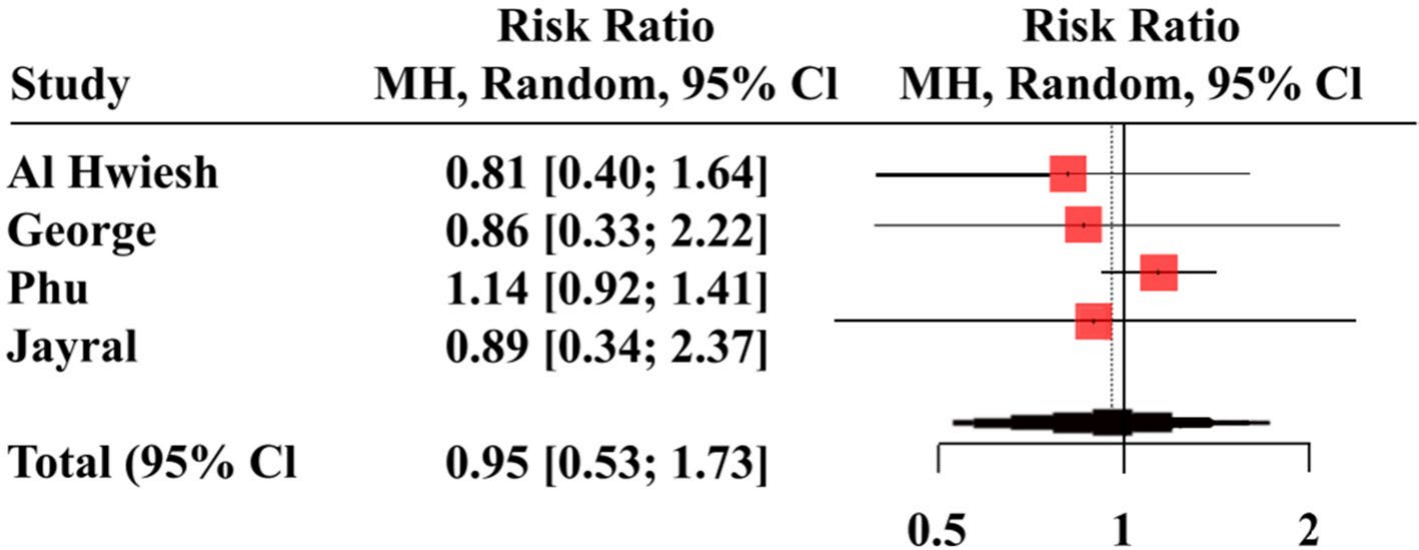
Leave-one-out sensitivity analysis comparing continuous renal replacement therapy (CRRT) and peritoneal dialysis (PD)

Leave-one-out sensitivity analysis showed that no single study had a disproportional effect on the pooled RR, which varied from 0.86 (95%CI 0.33, 2.22) when George et al. [28] was excluded to 1.14 (95%CI 0.92, 1.41) when Phu et al. [30] was excluded (Fig. 4**)**.

### PD vs. HD

**Fig. 5:**
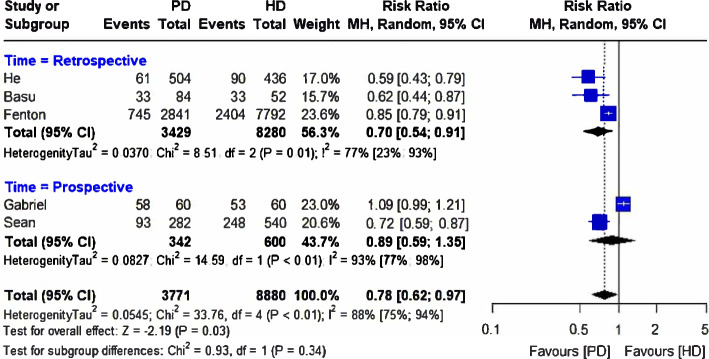
Forest plot comparing mortality rates between peritoneal dialysis (PD) and hemodialysis (HD)

**Fig. 6:**
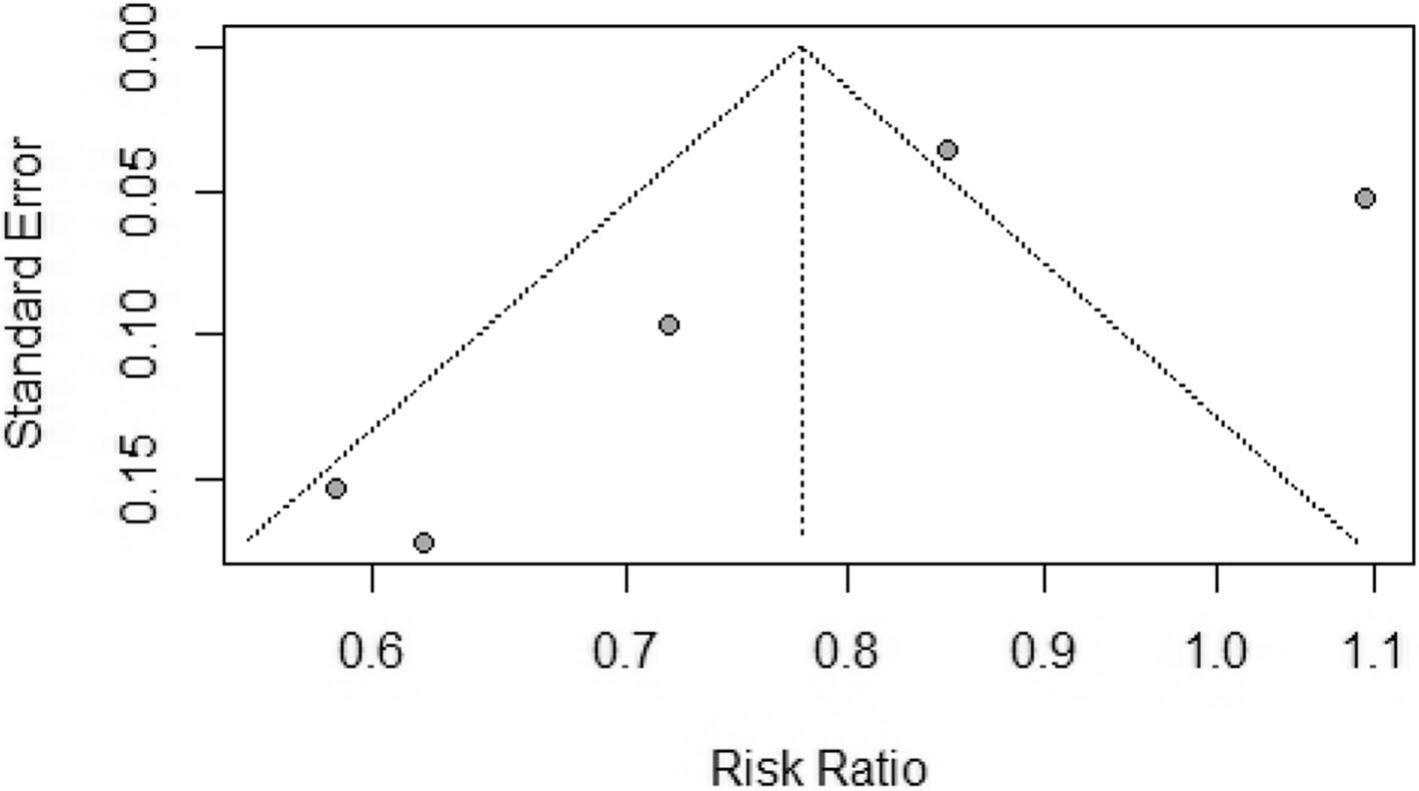
The funnel plot representing the publication bias between the selected studies comparing peritoneal Dialysis (PD) and hemodialysis (HD)

Another five studies with a large pooled participant size reported mortality rates with PD (*n* = 3,771) patients in the PD and HD (*n* = 8,880) treatment modalities [31–35]. Mortality risk significantly differed between the two groups favoring PD modality [RR = 0.78, (95%CI 0.62, 0.97), *p* = 0.03], although the studies had a high heterogeneity (*I*^2^ = 88%; *p* < 0.01) (Fig. 5). In the subgroup analysis, a difference in mortality rate was observed in retrospective [RR = 0.70, (95%CI 0.54, 0.97)] but not prospective [RR = 0.89, (95%CI 0.59, 1.35)] studies (Fig. 5). The funnel plot showing publication bias is presented in Fig. 6.

**Fig. 7:**
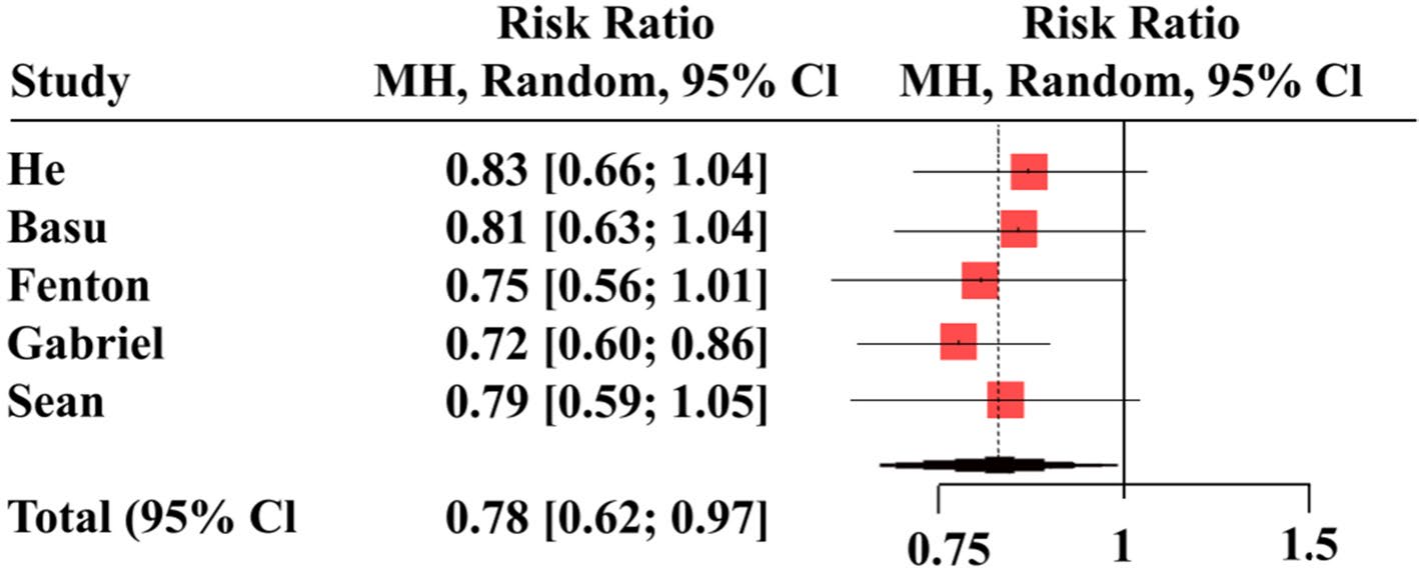
Leave-one-out sensitivity analysis comparing peritoneal dialysis (PD) and hemodialysis (HD)

The study by Basu et al. [31] had a different patient inclusion criteria than other studies and exclusively recruited pediatric patients aged one month to 16 years. In the leave-one-out sensitivity analysis, the pooled RR lost significance with the exclusion of Basu et al. [31] [RR = 0.81, (95%CI 0.63, 1.04)] as was the case with the exclusion of He et al. [35] [RR = 0.83, (95%CI 0.66, 1.04)], Fenton et al. [32] [RR = 0.75, (95%CI 0.56, 1.01)], and Murphy et al. [34][RR = 0.79, (95%CI 0.62, 0.97)] (Fig. 7). However, the pooled RR maintained significance favoring the PD modality with the exclusion of Gabriel et al. [33] [RR = 0.72, (95%CI 0.60, 0.86)] (Fig. 7).

### HD vs. CRRT

**Fig. 8:**
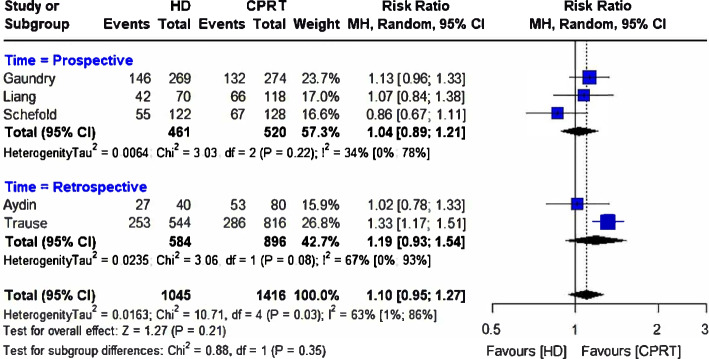
Forest plot comparing mortality rates between hemodialysis (HD) and continuous renal replacement therapy (CRRT)

**Fig. 9:**
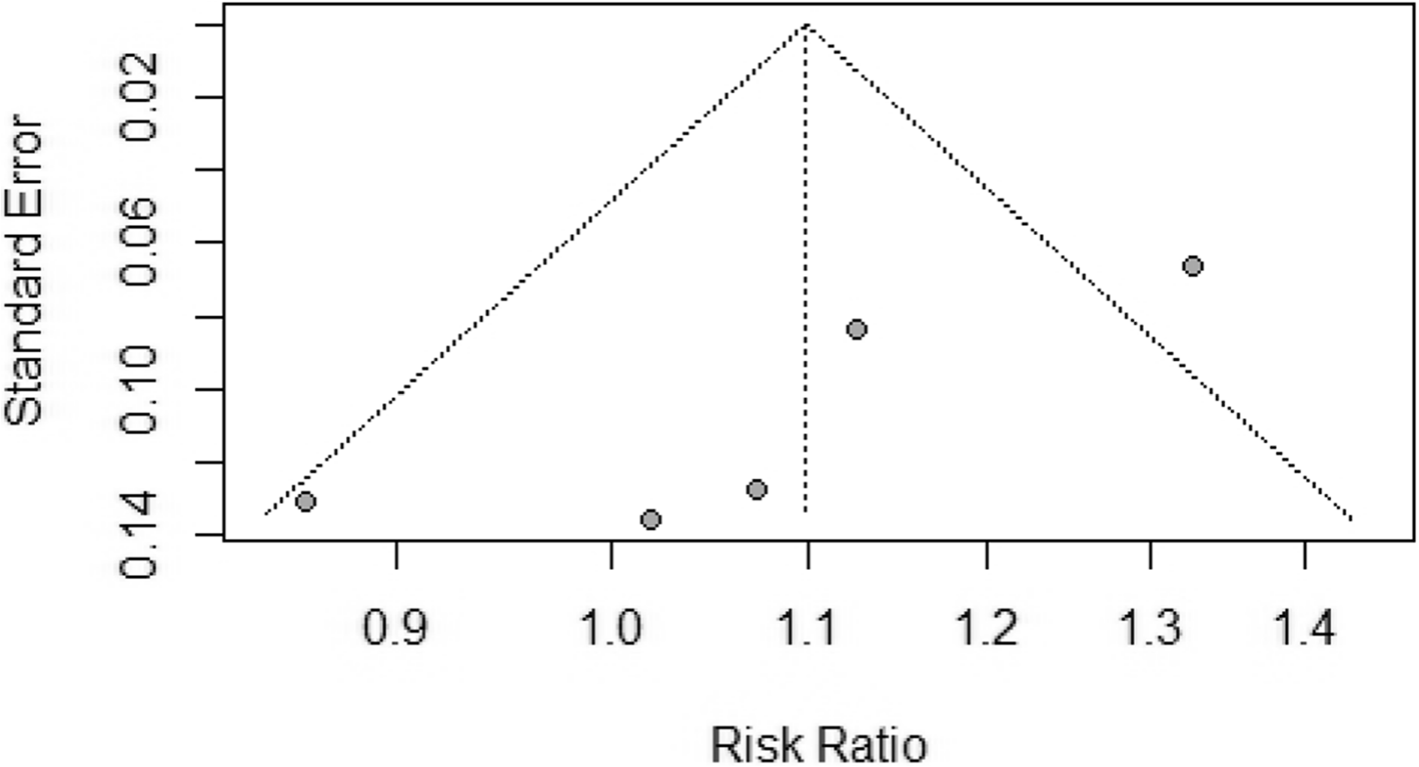
The funnel plot representing the publication bias between the selected studies comparing hemodialysis (HD) and continuous renal replacement therapy (CRRT)

Five studies provided mortality rates after CRRT and HD dialysis modalities with pooled participant sizes of 1,045 and 1,416, respectively [9, 36, 38–40]. The two treatment modalities did not differ in mortality risk [RR = 1.10, (95%CI 0.95, 1.27), *p* = 0.21] with moderate heterogeneity (*I*^2^ = 63%; *p* = 0.03) among the included studies (Fig. 8). The findings remained unchanged when prospective and retrospective studies were separately assessed in the subgroup analysis (Fig. 8). The funnel plot showing the publication bias is presented in Fig. 9.

**Fig. 10:**
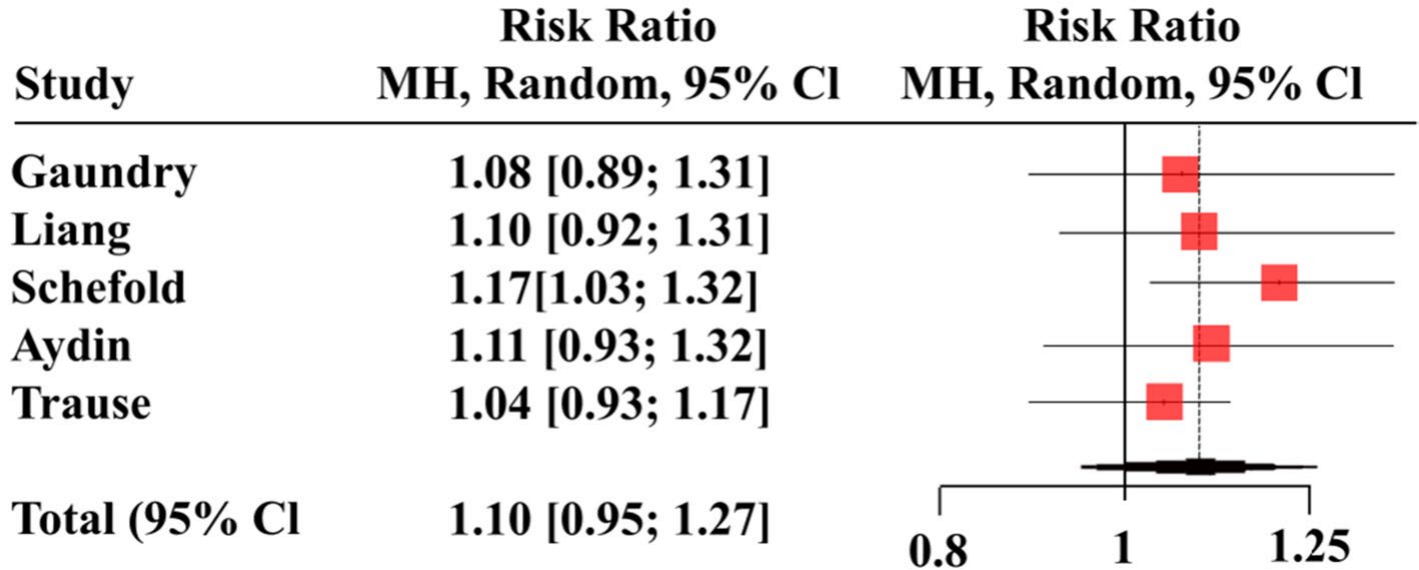
Leave-one-out sensitivity analysis comparing hemodialysis (HD) and continuous renal replacement therapy (CRRT)

In the leave-one-out sensitivity analysis, the exclusion of no single study significantly influenced the pooled RR (Fig. 10) despite the differences in the underlying patient condition among the included studies. Three studies involved patients with AKI [9, 36, 38], one involved patients with acute renal failure [39], and one was a sub-analysis of the patients who underwent at least one RRT session in the OUTCOMEREA multicenter cohort database; the inclusion criteria for the OUTCOMEREA was the receipt of mechanical ventilation for ≥ 48 h in an ICU setting [40].

### Risk of bias

**Fig. 11:**
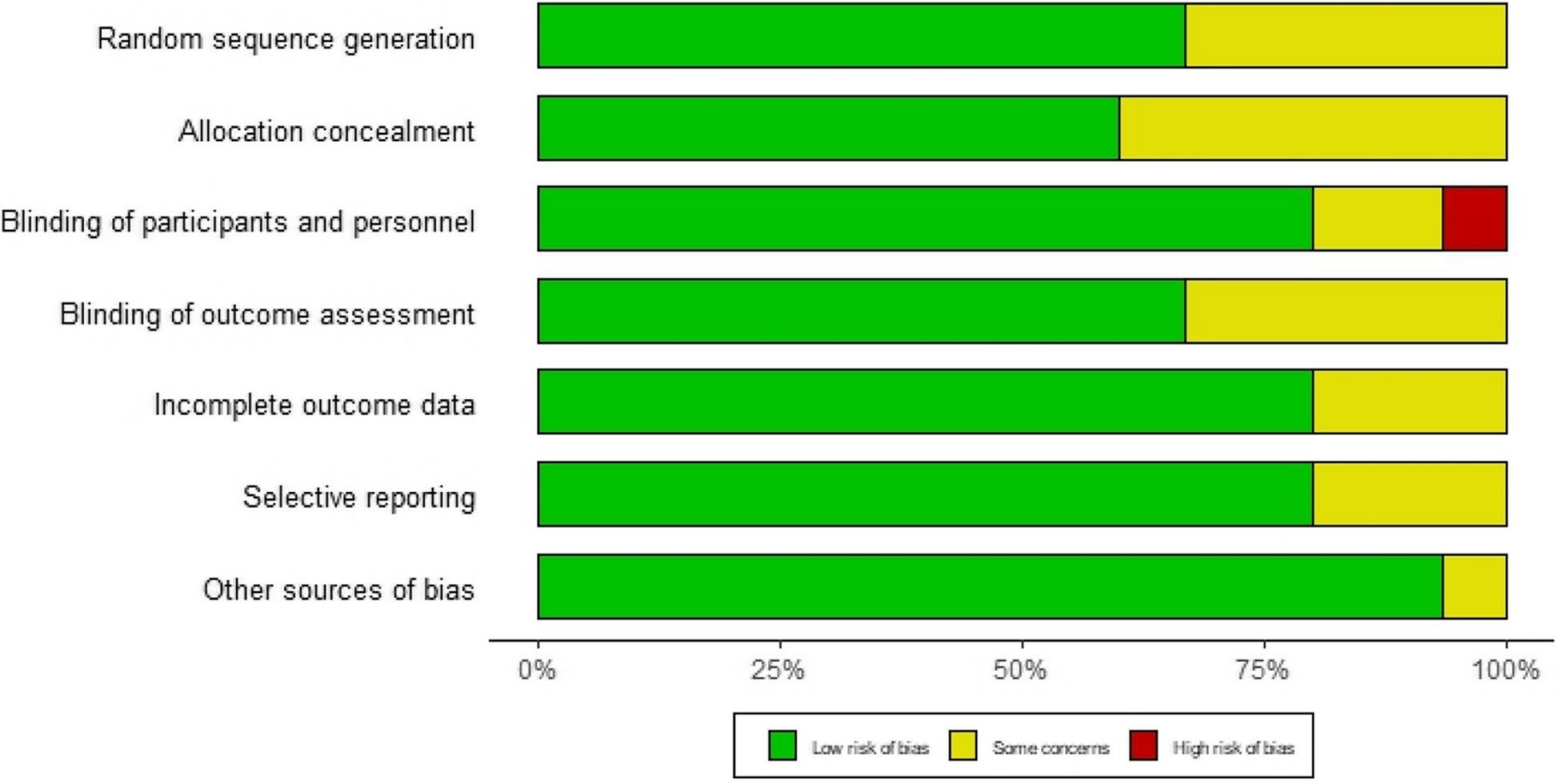
Risk of bias graph: review authors’ judgments about each risk of bias item presented as percentages across all included studies

**Fig. 12:**
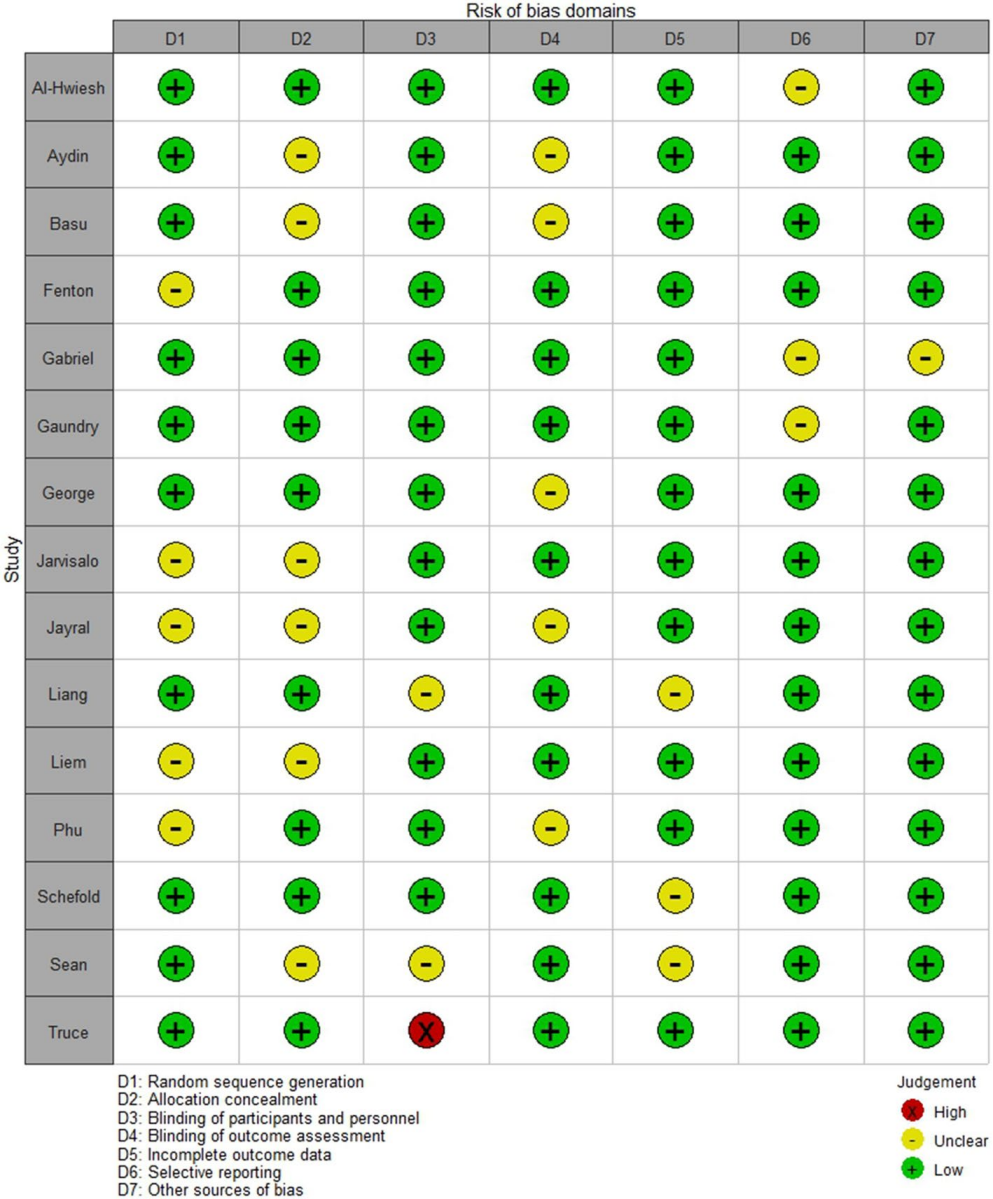
Risk of bias summary: review authors’ judgments about each risk of bias item for each included study

Figures 11 and 12 present a summary of the bias assessment risk. Five of the included had low risk of bias in six out of the seven assessed domains [9, 27, 28, 32, 39], six studies in five domains [30, 31, 33, 36–38], and two studies in four domains [29, 34]. The risk of bias was unclear in other domains in these 14 studies. However, in the study by Truche et al. [40], we detected a lack of blinding of participants and personnel as the study involved patients on mechanical ventilation in ICU settings, making blinding impractical.

## Discussion

Most prior meta-analytic studies comparing the mortality outcomes with hemodialysis modalities have been in patients with acute kidney injury. The most recent meta-analytic study by Ye et al. [12] included studies until May 2020 and demonstrated no significant differences in the mortality rates, recovery of kidney function, duration of renal support, or length of hospitalization with different dialytic modalities (CRRT vs. IHD, CCRT vs. PD, and IHD vs. PD) among patients with acute kidney injury with low certainty evidence due to high risk of bias and inconsistency between studies.

### CRRT vs. PD

Consistent with Ye et al. [12], we found no statistically significant differences in mortality among patients receiving CRRT and PD dialysis. However, there was considerable heterogeneity among the included studies. For instance, George et al. [28] reported numerically higher mortality rates among patients receiving CRRT than PD dialysis (84% vs. 72%), but the differences between the two modalities were not statistically significant. In contrast, Al-Hwiesh et al. [27] reported significantly different mortality rates between CCRT and PD (53.2% vs. 30.2%, *p* = 0.0028). However, Phu et al. [30] reported a significantly higher mortality rate among patients receiving PD (47%) than those receiving CRRT (15%, *p* = 0.005) in patients with infection-associated acute kidney failure.

We could not identify any meta-analytic study other than the one by Ye et al. [12] that compared mortality outcomes with CRRT vs. PD.

### PD vs. HD

However, we detect significant differences in mortality rates between patients receiving PD and HD favoring PD. This significance was lost in the leave-one-out sensitivity analysis with the exclusion of four studies, but the significance was maintained after the exclusion of Gabriel et al. [33]. Gabriel et al. [33] reported the highest mortality rates (58% vs. 53%) with both PD and HD modalities among the included studies. However, it only provided 1.6% of the participants in the pooled study population receiving PD and 0.7% of the participants in the pooled study population receiving HD. Like Gabriel et al. [33], Fenton et al. [32] also reported a non-significant difference in mortality rates in patients receiving PD or HD dialytic modalities (26.2% vs. 30.9%).

Nonetheless, Murphy et al. [34] reported higher mortality rates among patients receiving HD than those receiving PD therapy, albeit with a declining trend over six months of follow-up; mortality rates with HD were 45.9% at baseline, 41.3% at three months, and 39.8% at six months versus 33.3%, 35.6% and 33.8% with PD. Therefore, it is very likely that the differences in mortality trends diminish with duration of treatment modality.

In contrast to our findings, a meta-analysis by Xue et al. [41] demonstrated higher mortality risk with PD than HD in patients with ESRD. However, the study selectively included patients in whom diabetes was a cause of ESRD or significant comorbidity and from trials that reported intention to treat analysis and excluded as treated analysis [41]. Patients with diabetes have a higher risk of all-cause and cardiovascular mortality across the range of eGFR and ACR than those without diabetes [42]. Moreover, another meta-analytic study by Maruyama et al. [43] reported a high risk of bias due to inadequate control of confounding factors and high heterogeneity in the management of diabetes among observational studies reporting mortality outcomes with PD vs. HD in patients with concomitant ESRD and diabetes.

### HD vs. CRRT

Like the CRRT vs. PD comparison, we noted no significant difference in the mortality rates between patients receiving HD and CRRT therapy. Again, there was high heterogeneity among the included studies. Gaudry et al. [9] reported higher mortality in the CRRT group than in the HD group (HR 1.27, 95%CI 1.00 to 1.61) within the 60-day study duration. However, Liang et al. [38] reported numerically higher mortality rates with CRRT modalities than with HD therapy in severely ill patients at 90 days (60% vs. 55.9%) and 365 days (77.4% vs. 74.1%), albeit without statistical significance. Similarly, Yilmaz Aydin et al. [36] noted no statistical significance in the differences in mortality rates among patients in the HD and CRRT groups (66.3% vs. 67.5%).

Consistent with our findings, a meta-analysis of six randomized trials by Tonelli et al. [44] reported no association between CRRT and IHD and the rates of mortality, dialysis dependence, or recovery of kidney function in patients with acute renal failure, even after adjustment for disease severity at baseline. Furthermore, Zhang et al. [45] demonstrated that although extended daily HD in patients with acute kidney injury was associated with lower mortality than CRRT in observational studies, it did not differ in RCTs. Other outcomes, such as recovery of kidney function, fluid removal, or length of ICU stay, did not differ between the dialytic modalities in both observation studies and RCTs [45]. Similarly, an updated meta-analysis by Nash et al. [10] showed no difference in rates of hospital length of stay, in-hospital mortality, or dialysis dependence between critically ill patients undergoing CRRT or IHD.

### Limitations

The high heterogeneity among the included studies severely limits the generalizability of our findings. Therefore, underlying conditions such as worsening cardiovascular symptoms, diabetes, and age must be considered per patient before deciding on the dialytic modality [32, 34]. We did not have access to patient-level data to account for these variables. Furthermore, in addition to publication bias, the included studies may have residual bias, especially selection bias for the dialytic modality. Also, it is common for patients to change between dialytic modalities, which was not considered in most of the included studies.

Further, procedural details such as the type of catheter (rigid vs. flexible) or buffer (lactate acetate vs. bicarbonate) used [3] and the time of initiation of KRT may be potential confounders but were not reported by most of the included studies. Finally, this systematic review and meta-analysis was conducted to include different techniques of the three primary dialysis modalities, and, therefore, we did not search for studies reporting mortality outcomes with CRRT, HD, or PD techniques alone.

## Conclusion

The current evidence indicates that while patients receiving CRRT may have similar mortality risk compared to those receiving HD or PD, PD may be associated with lower mortality risk compared to HD. However, high heterogeneity among the included studies limits the generalizability of our findings. High-quality studies comparing mortality outcomes with different dialytic modalities in CKD are necessary for a more robust safety and efficacy evaluation.

## Data Availability

The datasets used and/or analyzed during the current study are available from the corresponding author on reasonable request.
